# A Rare Case of Community-Acquired Pneumonia Only Presenting With Diarrhea, Abdominal Pain, and Fever: A Case Report

**DOI:** 10.7759/cureus.44368

**Published:** 2023-08-30

**Authors:** Austin Miller, Punuru J Reddy, Derrick Randolph, Philip P Breton, Patrick Dickinson, Madeleine J Hyde

**Affiliations:** 1 Medicine, Alabama College of Osteopathic Medicine, Dothan, USA; 2 Internal Medicine, Decatur Morgan Hospital, Decatur, USA; 3 Family and Community Medicine, Decatur Morgan Hospital, Decatur, USA

**Keywords:** chronic watery diarrhea, chronic abdominal pain, outpatient clinic, legionella pneumonia, atypical pneumonia, legionnaires disease

## Abstract

Legionnaires' disease is an atypical pneumonia caused by Legionella pneumophila (*L. pneumophila)* pneumonia that features slow onset, nonproductive cough, fatigue, headache, sore throat, myalgias, and malaise. It can be difficult to diagnose, as it presents with extrapulmonary symptoms, and delay in treatment can be fatal. Here, we present the case of a previously healthy 32-year-old Caucasian male with Legionnaires disease who only presented to the clinic with abdominal pain and diarrhea. The patient did not have any pulmonary symptoms at the initial presentation. This presentation did not fit the diagnostic tools available for Legionnaires' disease, including a validated clinical prediction rule, which ruled out *L. pneumophila* infection with a sensitivity of 97% and a negative predictive value of 99.4%. Due to the complaint of abdominal pain, a flat/upright abdominal X-ray was ordered, which includes a chest X-ray. Upon analyzing the chest X-ray, a right lower lobe consolidation was identified, prompting an *L. pneumophila* urinary test to be added to the lab orders. This case represents the difficulties in diagnosing Legionnaires' disease due to the diverse clinical complexities of presentations, which may solely involve abdominal complaints.

## Introduction

Legionnaires' disease must be diagnosed correctly as it can have a fatality rate of 10% if left untreated [1]. In a retrospective study, Heath et al. reported that delaying antibiotic initiation after hospital admission and symptom onset were factors related to higher mortality in Legionella pneumophila (L. pneumophila) [2]. L. pneumophila has been associated with a 2-10% incidence of community-acquired pneumonia (CAP), with a higher incidence of severe CAP than common disease cases [2-5]. NNDSS data from 2014-2018, legionella incidence rates were twice as high in African Americans compared to Caucasians [6]. High-risk groups with more severe outcomes are elderly individuals (>50 years old), males, current or past smokers, persons with a history of alcohol abuse, and those with chronic comorbidities, including chronic obstructive pulmonary disease, diabetes, chronic kidney disease, and impaired cell-mediated immunity [7,8]. L. pneumonia presents with fever, cough, fatigue, and rales. Given these general symptoms, diagnosing Legionnaires disease can be difficult and/or challenging.

Tools to narrow down the differential diagnosis may not always be elucidating. X-ray findings can be inconsistent and vague, demonstrating single lobe consolidation, mostly in the lower lobes or multilobed opacities in an asymmetric pattern with rapid progression with or without a unilateral pleural effusion, but pleural effusions are common [9]. Also, diagnostic tests for this disease have low specificity for symptoms and sensitivity for diagnostic tests [10]. Bollinger et al. validated a clinical prediction rule to aid in the diagnosis, which includes dry cough, fever >39.4°C, C-reactive protein ≥187 mg/L, lactate dehydrogenase ≥225 mmol/L, hyponatremia (<133 mmol/L), and platelet counts <171 G/L. Fewer than two features rule out L. pneumophila infection with a sensitivity of 97% and a negative predictive value of 99.4% in CAP [11]. However, it has been noted that gastrointestinal symptoms such as nausea, emesis, and secretory diarrhea can raise clinical suspicion for Legionnaires' disease [12]. Patients who should be tested include those with moderate or severe CAP, those warranting hospitalization secondary to CAP, those with known or suspected exposure to Legionella with CAP symptoms, or those immunocompromised [12].

Pneumonias, in general, can be caused by bacteria, viruses, and fungi. Bacterial cases of pneumonia are frequently classified as either typical or atypical. Typical cases of pneumonia, which are commonly limited to the lungs, are mostly caused by Streptococcus pneumoniae (S. pneumoniae), Haemophilus influenzae (H. influenza), and Moraxella catarrhalis (M. catarrhalis) [13]. Atypical cases of pneumonia, which contrarily cause extrapulmonary manifestations, are commonly caused by Legionella spp, M. pneumoniae, C. pneumoniae, and Chlamydia psittaci [14]. Common viruses that cause cases of pneumonia include influenza, SARS-CoV-2, parainfluenza, respiratory syncytial virus, adenovirus, human metapneumovirus, Middle East respiratory syndrome coronavirus, and rhinovirus [15,16]. Fungi causes are rare and usually only present in immunocompromised patients [17].

L. pneumophila is a gram-negative bacteria found in freshwater lakes and rivers, whirlpools/hot tubs, swimming pools, showers, and air conditioning systems with contaminated condensed water [18]. Transmission occurs via inhalation of aerosols from contaminated water or soil [19]. Contamination of water and soil sources substantial enough to result in human infection may happen due to alterations in water flow or pressure, which disrupt biofilms, causing the release of bacteria into the reservoir [19]. Additionally, the bacteria can grow intracellularly within free-living amoeba, which may assist in the bacteria's transmissibility [18]. The most common route of transmission to humans is the propagation and colonization of anthropogenic freshwater reservoirs, which are most commonly air conditioner units and cooling towers. Many workers who operate and maintain these pieces of equipment are at a higher rate of L. pneumophila infection, especially if proper personal protective equipment (PPE) is not worn [18]. In the United States, Europe [18], and Australia, the reported incidence of Legionnaires' disease is 1.4-1.8 cases per 100,000 persons, with the highest reported incidence in New Zealand [6,19].

## Case presentation

**Figure 1:**
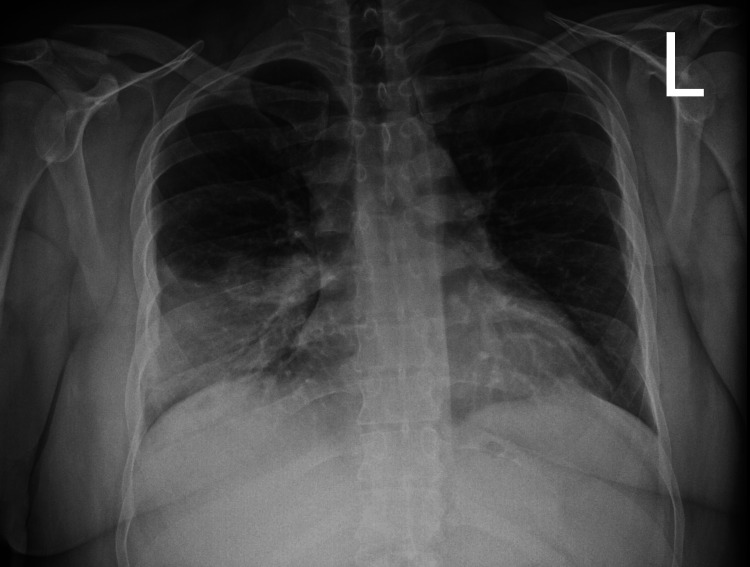
AP chest X-ray showing incidental lower lobe consolidation at initial presentation despite only complaining of abdominal pain and chronic diarrhea.

**Figure 2:**
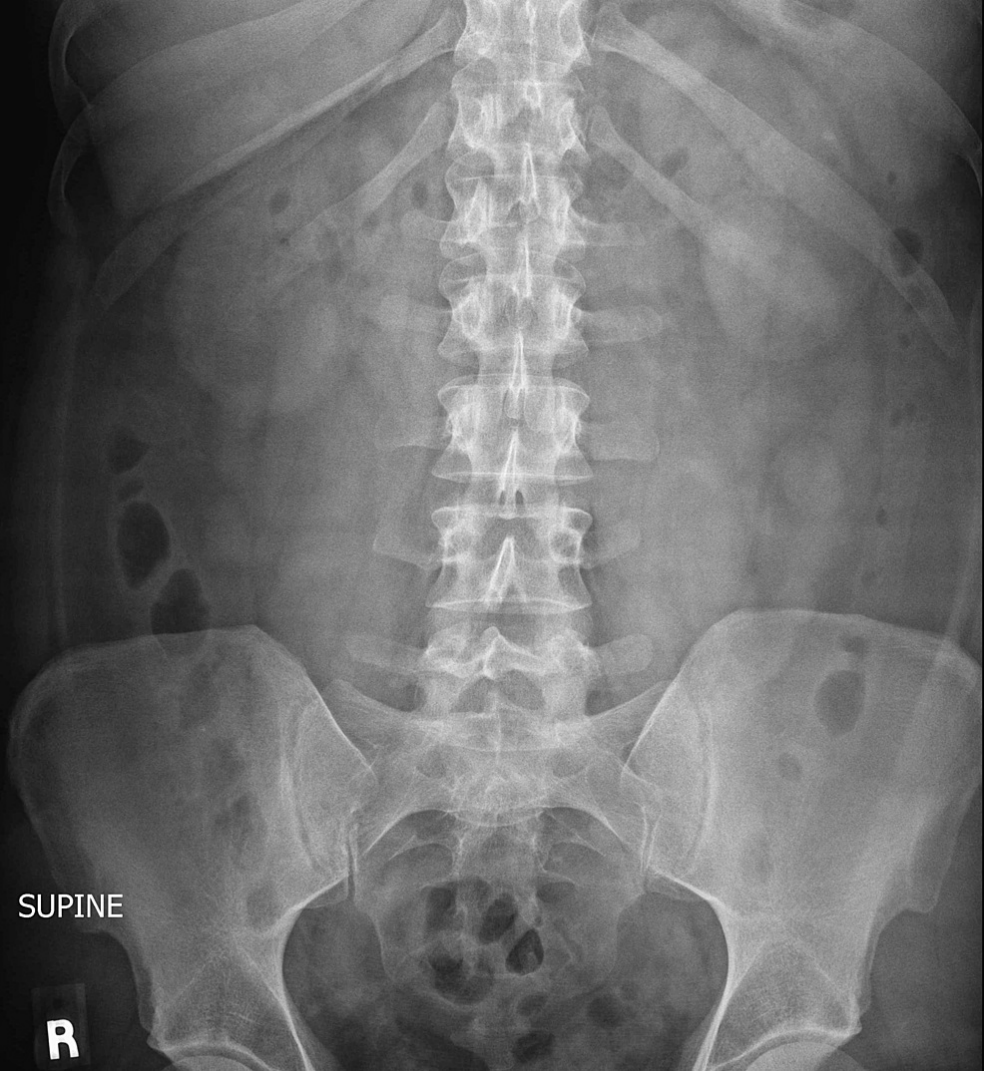
AP abdominal X-ray showing no bowel obstruction or other remarkable findings.

A previously healthy 32-year-old Caucasian male with a BMI of 33.4 presented to the outpatient clinic with a fever of 103.2 degrees Fahrenheit, abdominal pain, and intermittent diarrhea. Past medical history included asthma and no evidence of immunocompromising conditions. Social history did not involve smoking or alcohol use. Family medical history was reported as diabetes mellitus, cancer, and hypertension. The patient reported no travel history. The patient's occupation was simply noted on the intake form as a Technician. Based on the reported complaint, the physical exam focused on the abdomen, which was unremarkable. Flat/upright X-ray imaging was then ordered off the abdomen with the chest to rule out GI obstruction. Chest and abdominal X-ray in Figures 1-2 showed right lower lobe consolidation and no bowel obstructions. Following the X-ray findings, a physical exam of the lungs was conducted, finding bilateral wheezing and right unilateral rhonchi at mid and base lung fields. Labs showed normal sodium levels of 140 mEq/L, high WBC of 11.1 with high band neutrophils of 7% and low lymphocytes of 7%, low hematocrit (HCT) of 41.7%, high urine protein of 3+, high urobilinogen of 2, abnormal occult blood of 2+, high red blood cells in the urine 5-10/HPF, and bacteria of 1+.

**Figure 3:**
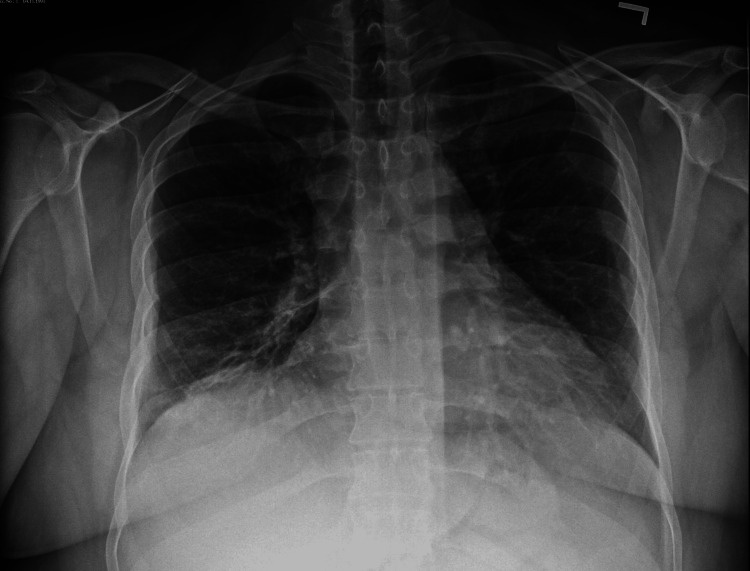
AP chest X-ray showing improved right lower lobe consolidation at a follow-up visit nine days later.

**Figure 4:**
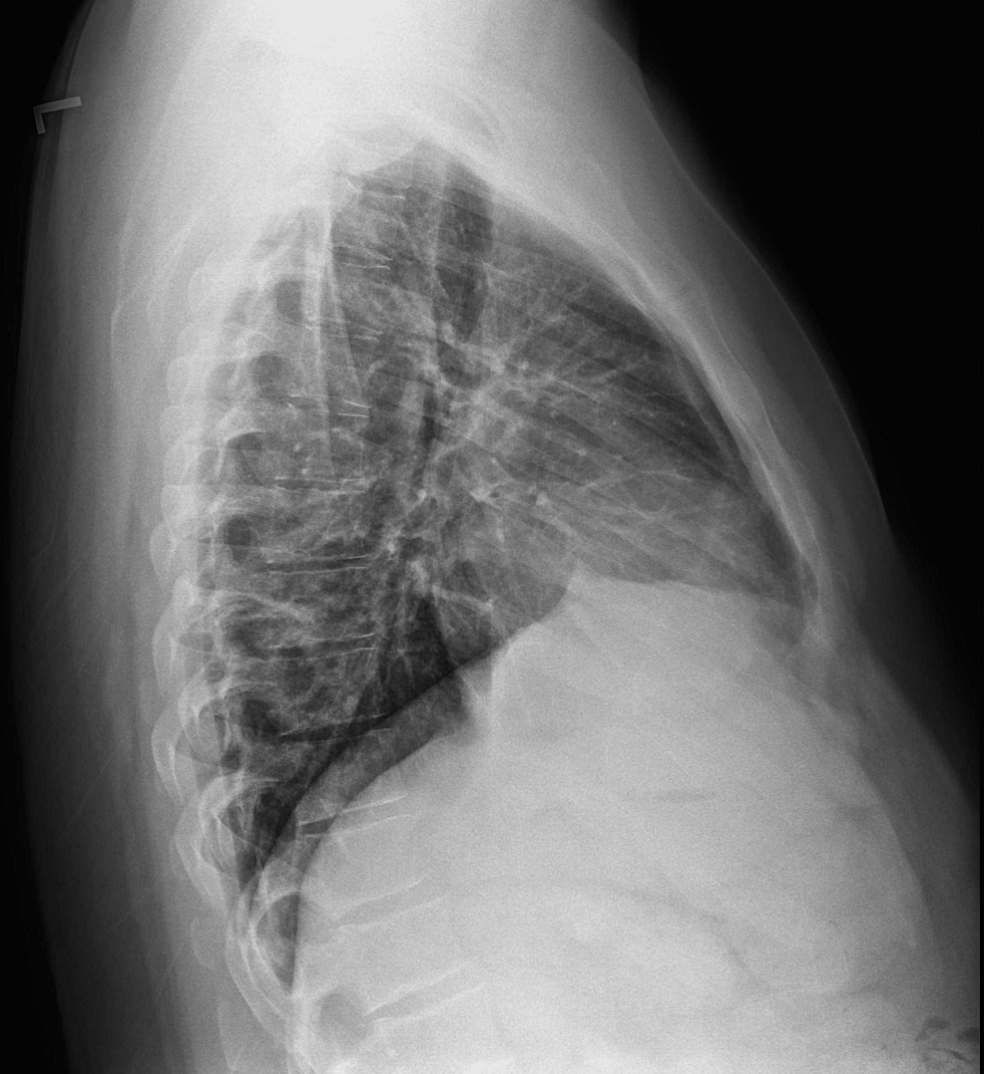
Lateral chest X-ray showing improvement of lower lobe consolidation at a follow-up visit nine days later.

COVID/SARS-CoV-2, flu A and B, and Respiratory Syncytial Virus (RSV) were all negative. The final assessment included atypical pneumonia, typical pneumonia, and aspiration pneumonia due to gastroesophageal reflux disease. The patient was suspected of having atypical pneumonia due to Legionella pneumophila based on his job occupation, which was later specified as a technician working on air conditioning units. Subsequently, an L. pneumophila urinary antigen test was ordered because there was no cough. This test confirmed that L. pneumophila was the cause of his pneumonia. Dexamethasone 4 mg and a cephalosporin, Rocephin, 500 mg injections were given in the office, and Levofloxacin 500 mg once per day, Duraflu 60-20-200- 325 mg four times per day, and dexamethasone 6 mg once per day were given for 10 days along with Promethazine-DM 6.25 mg/5mL 10 cc four times per day as needed. At the follow-up visit nine days later, both patient symptoms and chest X-ray showed improvement, as shown in Figures 3-4. 

## Discussion

Legionnaires' disease is difficult to diagnose despite tools like the validated clinical prediction rule available to help. Regarding the clinical prediction rule, L. pneumophila infection would have been ruled out with a 97% sensitivity and a negative predictive value of 99.4%. This patient's clinical features excluded hyponatremia, cough, and platelet abnormalities, while C-reactive protein and lactate dehydrogenase labs were not ordered. The only complaining symptoms were abdominal pain and diarrhea, without any pulmonary symptoms. Originally, the differential diagnoses included abdominal obstruction, gastroenteritis, food poisoning, appendicitis, inflammatory bowel disease, and urinary tract infection. With the incidence of Legionnaires' disease being low, the patient being young, and a nonsmoker, it was not initially included in the differential diagnosis. After chest x-ray findings, which prompted auscultation of the lungs, and inquiring into more specific details regarding the patient's occupation, which revealed that he was a technician for air conditioning units, the diagnosis of Legionnaires' disease was significantly considered. X-rays were only ordered to investigate the abdominal pain. Flat/upright X-ray imaging includes a chest X-ray, which demonstrated a right lower lobe infiltrate, as shown in Figure 1. This informed the differential diagnosis, including atypical pneumonia, typical pneumonia, and aspiration pneumonia from gastroesophageal reflux disease. The patient's occupation and nonspecific respiratory findings raised suspicion for L. pneumophila pneumonia. Without this incidental finding, pneumonia would not have been considered since the physical exam initially focused on the abdomen.

Additionally, the patient's obesity (BMI of 33.4) can make physical exams suboptimal and increase the likelihood that important auscultatory findings are not discovered, thereby hindering the clinical differential diagnosis. The patient was put on levofloxacin originally for x-ray and pulmonary findings before the L. pneumophila urinary antigen test results were known. This was unchanged due to the positive L. pneumophila urinary antigen test since levofloxacin and azithromycin are preferred for treatment with other fluoroquinolones and macrolides as alternatives. The patient demonstrated improvement in symptoms at the follow-up x-ray, as shown in Figure 2.

## Conclusions

L. pneumophila infections are easily misdiagnosed when not in an easily traceable outbreak. This case represents the difficulties in diagnosing Legionnaires' disease due to the diverse clinical complexities of presentations, which may initially only involve abdominal complaints. Available, validated tools like the clinical prediction rule can be helpful and cost-efficient, but they can also lead to inappropriately ruling out important pathologies in rare cases. Delays in antibiotic treatment in these cases can lead to higher chances of mortality.
